# Network Scale Modeling of Lymph Transport and Its Effective Pumping Parameters

**DOI:** 10.1371/journal.pone.0148384

**Published:** 2016-02-04

**Authors:** Samira Jamalian, Michael J. Davis, David C. Zawieja, James E. Moore

**Affiliations:** 1 Department of Bioengineering, Imperial College London, South Kensington Campus, London, United Kingdom; 2 Department of Pharmacology and Physiology, University of Missouri School of Medicine, Columbia, MO, United States of America; 3 Department of Systems Biology and Translational Medicine, Texas A&M Health Science Center, Temple, TX, United States of America; USF Health Morsani College of Medicine, UNITED STATES

## Abstract

The lymphatic system is an open-ended network of vessels that run in parallel to the blood circulation system. These vessels are present in almost all of the tissues of the body to remove excess fluid. Similar to blood vessels, lymphatic vessels are found in branched arrangements. Due to the complexity of experiments on lymphatic networks and the difficulty to control the important functional parameters in these setups, computational modeling becomes an effective and essential means of understanding lymphatic network pumping dynamics. Here we aimed to determine the effect of pumping coordination in branched network structures on the regulation of lymph flow. Lymphatic vessel networks were created by building upon our previous lumped-parameter model of lymphangions in series. In our network model, each vessel is itself divided into multiple lymphangions by lymphatic valves that help maintain forward flow. Vessel junctions are modeled by equating the pressures and balancing mass flows. Our results demonstrated that a 1.5 s rest-period between contractions optimizes the flow rate. A time delay between contractions of lymphangions at the junction of branches provided an advantage over synchronous pumping, but additional time delays within individual vessels only increased the flow rate for adverse pressure differences greater than 10.5 cmH_2_O. Additionally, we quantified the pumping capability of the system under increasing levels of steady transmural pressure and outflow pressure for different network sizes. We observed that peak flow rates normally occurred under transmural pressures between 2 to 4 cmH_2_O (for multiple pressure differences and network sizes). Networks with 10 lymphangions per vessel had the highest pumping capability under a wide range of adverse pressure differences. For favorable pressure differences, pumping was more efficient with fewer lymphangions. These findings are valuable for translating experimental measurements from the single lymphangion level to tissue and organ scales.

## Introduction

The balance of fluid and proteins in the body is maintained by transport of lymph from low or negative pressure tissue spaces through the lymph nodes and on to the subclavian vein near the heart (central venous pressure), against gravity (when standing upright) and without a central pump. This feat is accomplished mainly by contraction of lymphatic muscle cells in the walls of collecting lymphatic vessels. The forward propulsion of lymph is achieved via numerous unidirectional intraluminal valves throughout the system that segment the vessels into smaller contracting units (lymphangions) [1].

Lymph formation in the tissues and consistent flow throughout the lymphatic network is required to maintain tissue homeostasis and prevent edema [2]. Thus, it is important to identify the parameters that play major roles in efficient lymphatic pumping. Experimental studies on isolated lymphatic vessels have long aimed to determine the functional importance of such parameters at the lymphangion and tissue level. Since Reddy’s studies in 1970s, computational models have complemented the experimental evidence and provided additional insights where experimental measurements could not be performed [3, 4].

For lymphangions in a sequential series, the overall function and efficiency of lymph pumping is affected by coordination of the spontaneous, active contraction waves [5, 6]. Experimental observations of rat mesenteric lymphatics in situ indicate that contractions of adjacent lymphangions are coordinated along the vessels >80% of the time (i.e., same frequency with small time delay between contractions) with a slight preference for an orthograde propagation direction [5]. Mechanical blockade of the lymph bolus into the subsequent downstream lymphangion did not significantly alter the propagation characteristics, whereas a gap junction blocker significantly decreased coordination and propagation [5]. In vivo, both orthograde (in the flow direction of the valves) and retrograde (in the reverse direction) propagation along the length of the vessel have been observed in collecting mesenteric lymphatic vessels from bovine [7], rat [5] and guinea-pig [8]. Zawieja et al. reported similarly propagating contraction phenomena for branching vessels *in situ* [5]. Crow et al. found a greater likelihood of the upstream lymphangion constricting before the downstream lymphangion in guinea pigs mesentery (with a time delay about 0.5 s) [8]. They suggested that transmission of both mechanical and electrical signals between adjacent lymphangions could contribute to the coordination of contractile activity. Davis et al. observed both synchronous and conducted contractions with about 0.7 s time delay in isolated vessels from rat thoracic duct. In those preparations, the axial pressure difference modulated the time delay between contractions [9]. All of these studies emphasized the importance of quantifying the effect of propagation and coordination of spontaneous lymphatic contractions along vessel networks on lymphatic pumping. McHale also hypothesized that this coordination should be much more complex in a highly branched lymphatic network structure [7]. Previous computational models of multiple lymphangions in series have reported minimal change in average flow rate between forward and reverse propagating waves of contraction [10]. One study reported a small advantage in peak flow rate via the reverse propagation of the contraction wave [11]. In a lumped parameter model of lymphangions in series, Bertram et al. found that the time delay between contractions was more advantageous than the synchronization of contractions for pumping [6]. More recently, they reported that under adverse pressure differences lymphatic valve function could also be affected by the timing of contractions [12].

In addition to the effects of lymphangion contraction, external loads such as pressure and tissue tethering can modulate lymphatic pumping by influencing the effective transmural pressure. Mislin first described the stimulation of the lymph pump by pressure [1]. McHale et al. observed an increase in flow output of cannulated bovine mesenteric lymphatic vessels following a controlled rise in transmural pressure [13]. Gashev et al. reported that lymphatic vessels are very sensitive to transmural pressure and that most effective pumping is achieved under different levels of transmural pressures in lymphatics from different tissues; for example, optimum transmural pressure (resulting in maximum flow) for rat thoracic duct is ~1–3 cmH_2_O while that in the mesenteric lymphatics is ~5 cmH_2_O [14]. Eisenhoffer et al. also observed that cannulated bovine mesenteric lymphatic vessels exhibited optimal pumping over a certain range of transmural pressures. Additionally they observed that upon increase of outflow pressure, flow output decreases nonlinearly with pressure. They reported a lower variation in lymph flow in response to elevation of outflow pressure at lower transmural pressures [15]. We previously performed a parameter sensitivity study for a lumped parameter model of lymphangions in series that similarly demonstrated the existence of an optimum transmural pressure that maximized the flow rate as well as a non-linear pump function curve describing the flow rate drop as outflow pressure increased [16].

Due to the complexity of experimental measurements with the isolation and cannulation of lymphatic vessels, experimental data are limited in several functional aspects of the lymphatic system. Rigorous control over many of the parameters such as the timing of contractions, diastolic period, external pressure, inlet/outlet pressures (in situ), and network size is not possible experimentally. Additionally most of the experiments are performed for chains of lymphangions in series, while from anatomic observations we know that lymphatic networks are highly branched [17, 18]. Reddy 1974 was the first to attempt and develop a network model of post-nodal lymphatic vessels. The model however, did not include several key features of collecting lymphatic vessels such as highly non-linear pressure diameter relationship, realistic valve behavior, and phasic contractions. Here we have expanded our lumped parameter model of lymphangions in series [16] to a bifurcating network structure of collecting lymphatic vessels where each vessel contains multiple lymphangions. We have studied the collective network behavior (in terms of mean flow rate $\bar{Q}$) in response to variations in pressure difference (via outlet pressure *p*_b_), the size of the network (via number of lymphangions per vessel *n*_v_), transmural pressure (via *p*_e_), timing of contractions via variation of diastolic period for an individual lymphangion (*t*_r_), time delay between contraction of adjacent lymphangions (Δ*t*_v_), and time delay at the junctions (Δ*t*_g_).

Our results offer several novel predictions as to the function and pumping regulation of branched lymphatic networks and provide further insight into translation of experimental data from single lymphangions to the network scale.

## Materials and Methods

### Network structure

**Fig 1:**
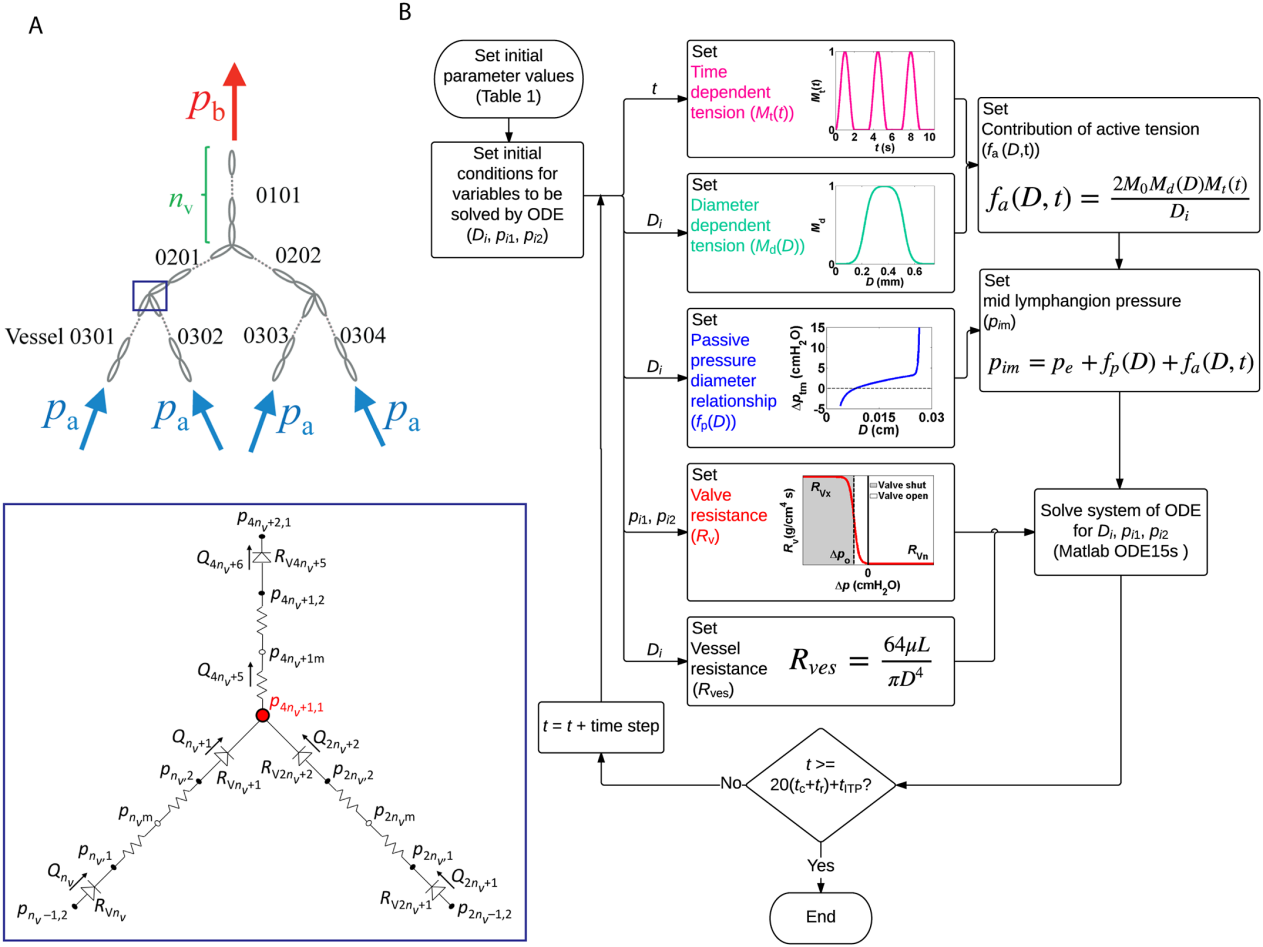
Network structure. (A) Top: Schematic view of the branching network structure with three generations of bifurcating vessels. The network pumps the fluid from inlets (*p*_a_) to the outlet (*p*_b_). The network is formed by seven vessels arranged in a bifurcating pattern (index 0101–0304). Each vessel is composed of multiple lymphangions and the number of lymphangions per vessel (*n*_v_) is the same for all the vessels in the network. Bottom: Electrical analogy schematic of one of the junctions in the network. (B) Diagram of the algorithm for solving the system of equations and the behavior of the main governing equations. *t*_ITP_ is the initial transient period after which a consistent periodic solution is achieved.

**Fig 2:**
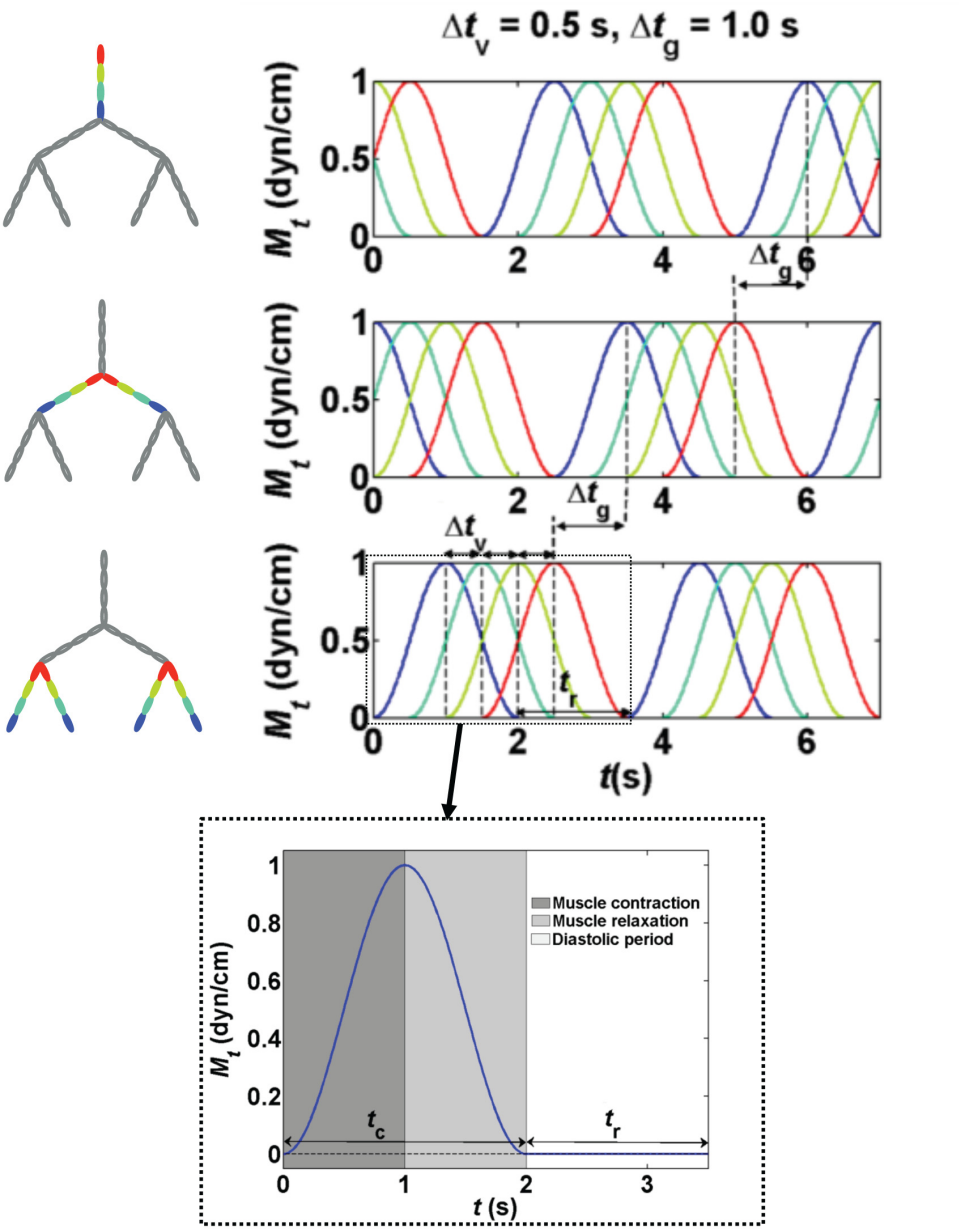
Timing of contractions. *M*_*t*_ (*t*) vs. time for branching network with four lymphangions per vessel (*n*_v_ = 4). Bottom panel: vessels 0301–0304. Mid panel: vessels 0201 and 0202. Top panel: vessel 0101. Δ*t*_v_ is the time delay between contractions of adjacent lymphangions in one vessel; Δ*t*_g_ is the time delay between two subsequent generations (at the junction). Specified time course for the contraction of an individual lymphangion (normalized *M*_*t*_ (*t*) vs. time) is presented.

We have developed a computational model for fluid transport in branching networks of lymphatic vessels in which each vessel is itself composed of a number of lymphangions *n*_v_ (Fig 1A). Individual lymphangions (along with their adjacent valves) are modelled using a lumped parameter approach, following the algorithm presented in Fig 1B and with more detail than in our previous studies [6, 16]. Briefly, equations of conservation of mass, conservation of momentum, and a force balance on the vessel wall are solved for each lymphangion, resulting in a nonlinear ordinary differential equation for diameter (*D*). The system of equations is then solved computationally, using the Matlab subroutine ode15s. The force balance on the vessel wall accounts for the force generated by active contraction of lymphatic muscle cells embedded in the wall of the lymphatic vessel as well as the passive behavior of the vessel. The tension generated by lymphatic muscle cells (*M*) is dependent on muscle length; therefore *M* varies with diameter of the vessel via a sigmoidal, diameter-dependent function (*M*_d_(*D*)). Muscle contraction and muscle relaxation are modelled using a time dependent sinusoidal function (*M*_t_(*t*)). Subsequent contractions of each lymphangion are separated by a period of relaxation (referred to as the diastolic period, duration *t*_r_). Time delays between contractions of adjacent lymphangions in one vessel (Δ*t*_v_) and in two subsequent generations (Δ*t*_g_) are implemented as well (Fig 2). The passive behavior of the vessel is dictated by the pressure diameter relationship obtained experimentally from isolated rat mesenteric lymphatic vessels [19]. Valves are modeled using a sigmoidal function with minimum resistance to forward flow which transitions to maximum resistance at a slightly negative pressure difference (Δ*p*_o_) to prevent backflow. This valve behavior was prescribed based on the experimental observations indicating that lymphatic valves are biased in the open position and there is a small amount of backflow before completely closing [20]. This is implemented via the small negative pressure difference Δ*p*_o_ required to close the valve (Δ*p*_o_ remains constant in all situations, whereas other studies have explored manipulations of this parameter and its effect on pumping of small chains of lymphangions [12, 19, 21]). The baseline values of the parameters of the model are presented in Table 1. The equations of the model are as follows:

Conservation of mass:
  $$Q_{i+1}=Q_{i}-\frac{\pi D_{i}L_{i}}{2}\frac{dD_{i}}{dt}.$$
Conservation of momentum:
  $$p_{i-1,2}-p_{i1}=R_{\text{V}i}\;Q_{i};\;p_{i1}-p_{i\text{m}}=R_{\text{ves}i\;}Q_{i};$$
  $$p_{i\text{m}}-p_{i2}=R_{\text{ves}i}\;Q_{i+1};\;p_{i2}-p_{i+1,1}=R_{\text{V}i+1}\;Q_{i+1},$$
  where **$R_{\text{V}i}=R_{\text{V}i}\left(p_{i-1,2}-p_{i1}\right)\;$** and **$R_{\text{V}i+1}=R_{\text{V}i+1}\left(p_{i2}-p_{i+1,1}\right)$**. With $R_{\text{V}i}=R_{\text{Vn}}+R_{\text{Vx}}\left(\frac{1}{1+e^{-s_{\text{f}}\left(\Delta p-\Delta p_{\text{f}}\right)}}+\frac{1}{1+e^{s_{\text{o}}\left(\Delta p-\Delta p_{\text{o}}\right)}}-1\right)$, and $R_{\text{ves}i}=\frac{64\mu L_{i}}{\pi D_{i}^{4}}$.
Pressure at the junctions (e.g., junction in Fig 1A):
  $$Q_{4n_{v}+5}=Q_{2n_{v}+2}+Q_{n_{v}+1},\;p_{4n_{v}+1,1}-p_{4n_{v}+1\text{m}}=R_{\text{ves}4n_{v}+1}\;Q_{4n_{v}+5};$$
  Thus $\frac{p_{4n_{v}+1,1}-p_{4n_{v}+1m}}{R_{\text{ves}4n_{v}+1}}=\frac{p_{2n_{v}m}-p_{4n_{v}+1,1}}{R_{\text{ves}2n_{v}}+R_{\text{V}2n_{v}+2}}+\frac{p_{n_{v}\text{m}}-p_{4n_{v}+1,1}}{R_{\text{ves}n_{v}}+R_{\text{V}n_{v}+1}}$.
Vessel wall force balance:
  $$\Delta p_{tm}=p_{im}-p_{e}=f_{p}(D)+f_{a}(D,t);$$
  $$f_{p}(D)=P_{d}[c_{1}{\left(\frac{D_{i}}{c_{9}}-c_{2}\right)}^{2}+c_{3}\text{exp}(c_{4}(\frac{D_{i}}{c_{9}}-c_{5}))+c_{6}+c_{7}(\frac{D_{i}}{c_{9}}–c_{8})+c_{10}{\left(\frac{c_{9}}{D_{i}}\right)}^{3}],$$
  With
  Pd=732,Dd=0.00845c1=–2.34457751,c2=1.1262924, c3=3.76013762, c4=79.991135c5=1.0028029,c6=1.59133174, c7=3.69692633, c8=0.20699868c9=Ddc11,c10=–0.0180867408, c11 = 0.32538081
  $f_{a}(D,t)=\frac{2M(D,t)}{D_{i}}$ and $M(D,t)=M_{0}\times M_{d}(D)\times M_{t}(t)$.
  $$M_{t}(t)=\left(1–\text{cos}\left(2\pi f(t–t_{c})\right)\right)/2,$$
  where *t*_c_ ≤ *t* ≤ *t*_c_ + 1/*f* and *t*_c_ defines the start of a contraction; the following contraction begins at *t*_c_ + 1/*f + t*_r_.
  $$M_{\text{d}}(D)=\frac{1}{1+e^{-s_{\text{d}}\left(D_{i}-D_{\text{a}}\right)}}+\frac{1}{1+e^{s_{\text{d}}\left(D_{i}–D_{\text{a}}\right)}}-1,$$
  with $s_{d}=\frac{3.25}{D_{d}},D_{a}=\text{0.85}c_{9},D_{b}=\text{2}c_{9}$.

**Table 1:** Parameters used in the numerical model, including their definition and baseline value.

Description	Parameter	Value	Units
Valve parameters			
Valve failure pressure	Δ*p*_f_	–18.4	cmH_2_O
Valve failure slope	*s*_f_	0.049	cm^2^/dyn
Minimum valve resistance	*R*_Vn_	0.5 × 10^6^	g/(cm^4^ s)
Maximum valve resistance	*R*_Vx_	9.9× 10^9^	g/(cm^4^ s)
Valve opening pressure	Δ*p*_o_	–15	dyn/cm^2^
Valve opening slope	*s*_o_	0.4	cm^2^/dyn
Pressure difference across valve *i*	Δ*p*	*p*_i − 1,2_ − *p*_i,1_	cm^2^/dyn
Non-valve parameters			
Fluid viscosity	*μ*	0.01	g/(cm s)
Lymphangion length	*L*	0.3	cm
Number of lymphangions per vessel	*n*_v_	4	-
Pressure constant in vessel wall force balance relation	*P*_d_	732	dyn/cm^2^
Diameter constant in vessel wall force balance relation	*D*_d_	0.00845	cm
Active tension	*M*_0_	250	dyn/cm
Contraction frequency	*f*	0.5	Hz
Contraction period	*t*_c_ (1/*f*)	2.0	s
Diastolic period	*t*_r_	1.5	s
Inter-lymphangion time difference	(via Δ*t*_g_, Δ*t*_v_)	0.5	s
External pressure	*p*_e_	2	cmH_2_O
Inlet pressure	*p*_a_	6	cmH_2_O
Outlet pressure	*p*_b_	9	cmH_2_O
Pressure difference between inlet and outlet of the network	Δ*P*	*p*_b_− *p*_a_	cmH_2_O

To construct the computational model of a tree-like network, individual lymphangions were added first in series then arranged to create branching network structures. Vessel junctions in the network were modeled by equating the pressures and satisfying conservation of mass. The model predictions for a network with three generations of bifurcated vessels (Fig 1) are presented here. To facilitate a systematic analysis, model parameters describing individual lymphangions (valve and non-valve parameters, Table 1) and the number of lymphangions per vessel (*n*_v_) were equal throughout the network (unless stated otherwise) and remained constant during each simulation except for the parameters studied (further details in the next section). The time averaged flow rate of the outlet lymphangion ($\bar{Q}$) in the network was chosen to represent the system output.

### Parameter variations

The impact of network size on its pumping capability was investigated by generating pump function curves for bifurcated networks with 1 to 14 lymphangions per vessel (*n*_v_ = 1–14). In these studies lymphangion length (*L*) remained constant; as a result variation of *n*_v_ contributes to longer vessels. All the other network geometry parameters remained constant at their baseline values. Based on these pump function curves we chose four lymphangions per vessel (*n*_v_ = 4) as our baseline network size. We investigated the flow rate response to manipulations in *p*_out_ and transmural pressure (Δ*p*_tm_), via variation of external pressure (*p*_e_), for three network sizes of 2, 4, and 8 lymphangions per vessel.

Pumping coordination within the network is determined by 1) the diastolic period and 2) time delay between contractions of adjacent lymphangions. In the branching network structure we recognize two categories of time delays: the time delay between contraction of adjacent lymphangions in one vessel (Δ*t*_v_) and between two subsequent generations (Δ*t*_g_: the time delay at the junction) (Fig 2).

Diastolic periods on the order of 9 s have been observed in collecting rat mesenteric lymphatic vessels [22, 23]. Here we obtained pump function curves for bifurcating networks with *n*_v_ = 4 when *t*_r_ increased by steps of 0.5 s from 0 to 3.5 s. Additionally, we determined the relative importance of time delay within vessels and time delay at the junction for effective pumping under varying levels of outlet pressure. In studying lymphatic branch points, Zawieja et al. observed little difference in the propagation of contraction waves between lymphangions in a parent vessel (primary vessel segment) vs. at the junctions, suggesting that under physiologic conditions Δ*t*_g_ = Δ*t*_v_ is a valid assumption [5]. Based on this knowledge, we investigated the impact of the time delay between contractions when Δ*t*_v_ = Δ*t*_g_ under different external pressures and pressure differences. Δ*t*_v_ = Δ*t*_g_ was incrementally increased from 0 to *t*_c_ + *t*_r_. Time delays smaller than half of total cycle time (Δ*t*_g_ = Δ*t*_v_ < ½(*t*_c_ + *t*_r_) = 1.75 s) represent the forward propagating contraction wave and larger time delays are equivalent to the reverse propagating waves (Fig 2). For reverse travelling waves the equivalent time delay in the reverse direction can be calculated as *t*_c_ + *t*_r_–(Δ*t*_g_ = Δ*t*_v_). For example, Δ*t*_g_ = Δ*t*_v_ = 3.25 s is equivalent of 0.25 s time delay in the reverse direction, when total cycle time is 3.5 s. The simulation conditions for all the cases studied here are summarized in Table 2; the parameters not listed in this table take their baseline values (see Table 1).

**Table 2:** Simulation conditions for all the cases studied here.

	Effect of *n*_v_	Effect of Δ*p*_tm_	Effect of *t*_r_				Effect of Δ*t*_g_ and Δ*t*_v_			
*n*_v_	1 − 14	2, 4, 8	2	4		8	4			
*p*_a_ (cmH_2_O)	6	6	6	6	6	6	6			
*p*_b_ (cmH_2_O)	3 − 41	9, 16, 22	9	9	3 − 36	9	9	3 − 36		-3, 0, 6, 12, 18, 24
*p*_e_ (cmH_2_O)	2	1 − 5	2	2	2	2	1, 2, 3, 4	2,3	2	2
*t*_r_ (s)	1.5	1.5	0 − 5	0 − 5	0 − 3.50.5 steps	0 − 5	1.5	1.5	1.5	1.5
*t*_c_ (s)	2	2	2	1.25, 2, 2.5	2	2	2	2	2	2
Δ*t*_g_ (s)	0.5	0.5	0.5	0, 0.5	0.5	0.5	0 − 3.5	0, 0.5, 0.5	0, 0.5, 1, 1.5, 1.75 (forward/reverse)	0 − 3.5
Δ*t*_v_ (s)	0.5	0.5	0.5	0, 0.5	0.5	0.5	Δ*t*_g_ = Δ*t*_v_	0, 0, 0.5	Δ*t*_g_ = Δ*t*_v_	Δ*t*_g_ = Δ*t*_v_
Fig #	3A	4	5A	5A	5B	5A	6B	6A	7A	7B

## Results

### Number of lymphangions per vessel

**Fig 3:**
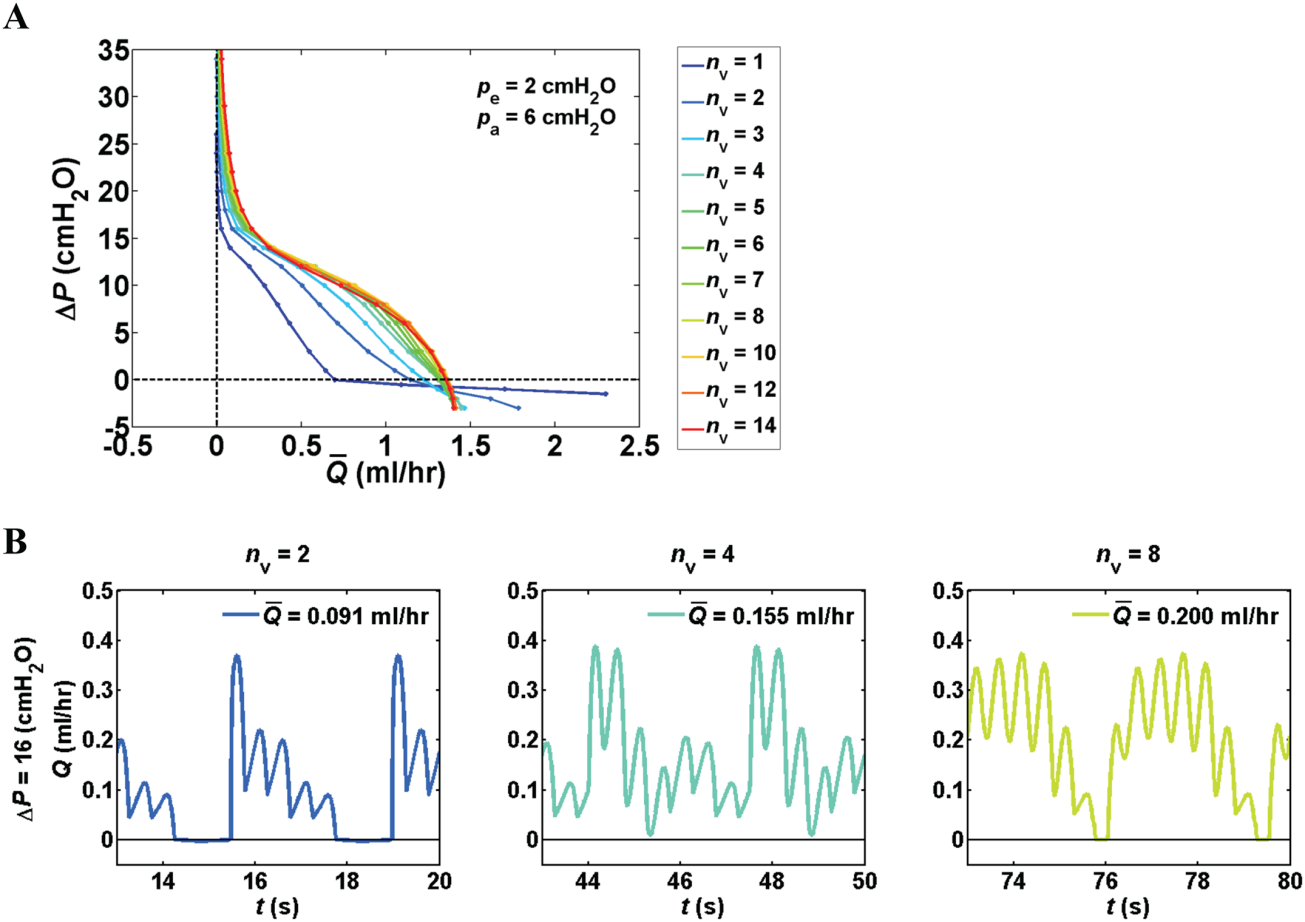
Effect of number of number of lymphangions (*n*_v_). (A) Pump function curves for branching networks with *n*_v_ = 1–14 (associated *L*_s_ = 0.3–4.2 cm). Under favorable pressure differences (Δ*P* < 0) maximum flow rate was achieved with *n*_v_ = 1 because additional valves increased the resistance. Flow rate increased with *n*_v_ when Δ*P* > 16 cmH_2_O. (B) Flow waveforms versus time for networks with *n*_v_ = 2, 4, and 8 at Δ*P* = 16 cmH_2_O. Networks with larger *n*_v_ benefit from additional valves to maintain the pressures required to keep downstream valves open in the presence of an adverse Δ*P*.

Pump function curves of networks with one to fourteen lymphangions per vessel showed that networks with smaller *n*_v_ were able to generate greater flow under favorable pressure differences. Specifically, at Δ*P* = –3 cmH_2_O (Δ*P* < 0 represents a favorable axial pressure difference), the network with a single lymphangion in each vessel had the highest flow rate of 3.29 ml/hr (this curve is cut off in the figure at Δ*P* = –1.5 cmH_2_O and flow rate of 2.3 ml/hr to allow for better representation of the other curves.), whereas the lowest flow rate of 1.40 ml/hr occurred for the network with *n*_v_ = 14. Under adverse pressure differences up to 10 cmH_2_O, *n*_v_ = 10 was optimal and resulted in flow rates of 1.37 ml/hr at 0 pressure difference and 0.82 ml/hr at Δ*P* = 10 cmH_2_O. At Δ*P* = 12 and 14 cmH_2_O peak flow rates of 0.58 and 0.33 ml/hr were achieved with 6 and 8 lymphangions per vessel, respectively, but the advantage over *n*_v_ = 10 was less than 2%. Using *n*_v_ = 10 produced the highest flow rate (0.21 ml/hr) at Δ*P* = 16 cmH_2_O (Fig 3A).

Higher flow rates were achieved with more lymphangions under larger adverse pressure differences (Δ*P* >16 cmH_2_O). For example at Δ*P* = 18 cmH_2_O, the network with a single lymphangion in each vessel produced only $\bar{Q}$ = 0.012 ml/hr, yet the network with *n*_v_ = 14 was able to produce 12× more flow. Flow waveforms for networks of 2, 4, and 8 lymphangions per vessel revealed that the more efficient pumping under high adverse pressure differences in networks with larger *n*_v_ results from the large number of pumping elements that build up the pressures required for the valves to remain open for a longer period in the face of an adverse pressure difference (Fig 3B).

### Effect of external pressure

**Fig 4:**
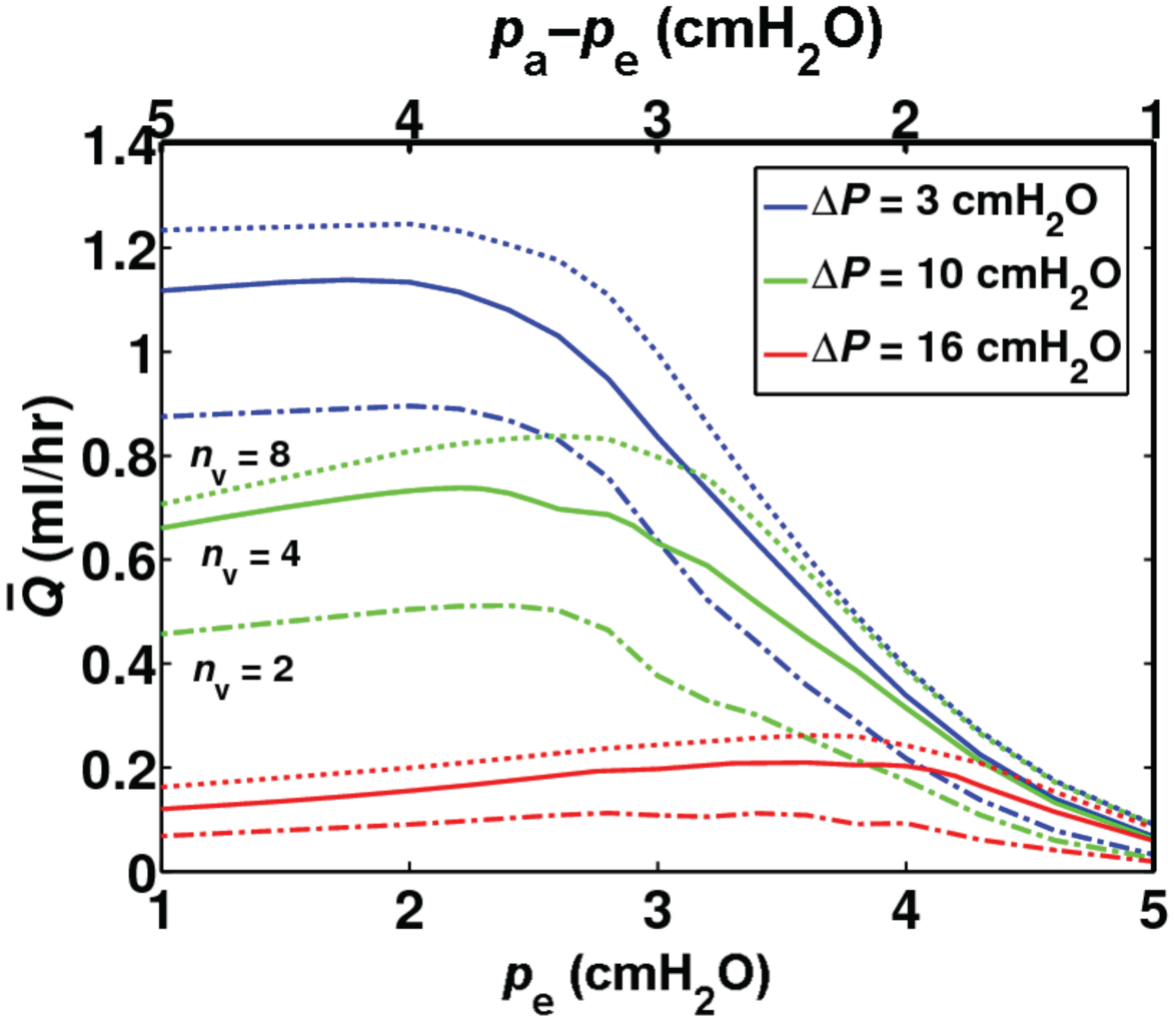
Effect of transmural pressure (via *p*_e_).$\bar{Q}$ vs. external pressure at Δ*P* = 3, 10, 16 cmH_2_O for branching networks with *n*_v_ = 2 (dot-dashed), *n*_v_ = 4 (solid), and *n*_v_ = 8 (dotted). The top axis indicates transmural pressure. Peak flow rate occurred at higher values of *p*_e_as pressure difference increased. Inlet pressure *p*_a_ was constant and equal to 6 cmH_2_O.

The flow rate was maximized at certain values of transmural pressure, ranging from 2 to 4 cmH_2_O, depending on Δ*P* (Fig 4). At lower values of transmural pressure (corresponding to high external pressure in this figure), productive pumping was essentially eliminated. Transmural pressures higher than this range (low external pressure) minimally affected the flow output. These findings correspond well with previous experimental observations on the heterogeneity of lymphatic pumps from different regions of the body [14]. Peak flow rate occurred at higher transmural pressures as pressure load increased. At Δ*P* = 3 cmH_2_O, flow rate was maximized at *p*_e_ ≈ 2 cmH_2_O. External pressures resulting in peak flow rate ranged between 2.2 and 2.6 cmH_2_O at Δ*P* = 10 cmH_2_O for all network sizes tested. At Δ*P* = 16 cmH_2_O, maximum $\bar{Q}$ occurred when *p*_e_ ≈ 2.8 cmH_2_O for *n*_v_ = 2, and *p*_e_ ≈ 3.6 cmH_2_O for networks with four and eight lymphangions per vessel. For *n*_v_ = 2, peak flow rates of 0.90, 0.51, and 0.11 ml/hr were achieved at Δ*P* = 3, 10, and 16 cmH_2_O, respectively. Peak $\bar{Q}$ increased to 0.14, 0.74, 0.21 ml/hr for *n*_v_ = 4, and 1.25, 0.84, and 0.26 ml/hr for *n*_v_ = 8. In these studies the inlet pressures were always set at 6 cmH_2_O, thus optimum pumping was achieved under external pressures lower than the inlet pressure (*p*_e_<*p*_a_).

### Pumping coordination in branching networks

#### Effect of diastolic period

**Fig 5:**
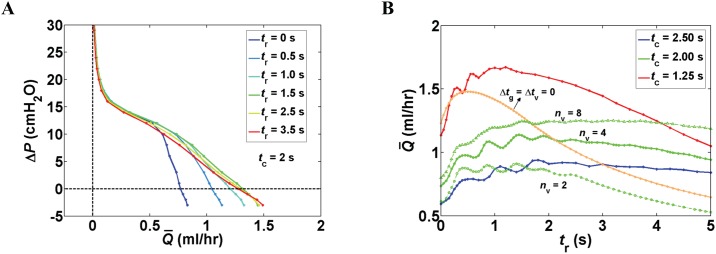
Effect of diastolic period (*t*_r_). (A) Pump function curves for a branching network with *n*_v_ = 4 as *t*_r_ increased from 0 to 3.5 s. Diastolic period was advantageous to pumping, particularly at low Δ*P*, because relaxed vessels present less impedance to incoming flow. (B) Combined effect of diastolic period, contraction period (*t*_c_), and number of lymphangions per vessel (*n*_v_) at Δ*P* = 3 cmH_2_O.

Pump function curves of networks with four lymphangions per vessel demonstrated that the addition of a diastolic period (*t*_r_) between contractions is advantageous for pumping. Under favorable pressure differences, we observed the highest flow rate of 1.49 ml/hr with the longest *t*_r_ (= 3.5 s here), whereas maximum pumping output was achieved with *t*_r_ = 1.5 s under adverse pressure differences. When Δ*P* > 10 cmH_2_O, little advantage was gained with variation of *t*_r_ (Fig 5A). Simultaneous variation of *n*_v_ and *t*_r_ revealed that longer diastolic periods were required to maximize $\bar{Q}$ in larger networks. At Δ*P* = 3 cmH_2_O, maximal flow rate occurred with longer diastolic periods as the number of lymphangions increased (with higher flow rates at larger *n*_v_). The changing pattern observed here is the result of interplay between variation in diastolic period and time delay between contractions of adjacent lymphangions (Δ*t*_v_ = Δ*t*_g_ = 0.5 s). When Δ*t*_v_ = Δ*t*_g_ = 0 s (*n*_v_ = 4), flow rate peaked at *t*_r_ = 0.5 s and continuously dropped for longer diastolic periods. At this pressure, peak flow rate achieved with synchronous pumping yielded values close to the maximum outflow of the network with *n*_v_ = 4 and *t*_c_ = 1.25 s. In the branching network structures with four lymphangions per vessel, simultaneous variation of *t*_r_ and period of contraction (*t*_c_ *=* 1/*f*) at Δ*P* = 3cmH_2_O showed that faster contractions (shorter period of contraction, higher frequency) reduces the diastolic period required to achieve peak $\bar{Q}$. Peak flow rates were observed when diastolic period was 1.8, 1.5, and 1.2 s, for contraction periods of 2.5, 2.0, and 1.25 s, respectively. Peak flow rate was higher when vessel contracted at a higher frequency (Fig 5B).

#### Effect of time delay between contractions of adjacent lymphangions

**Fig 6:**
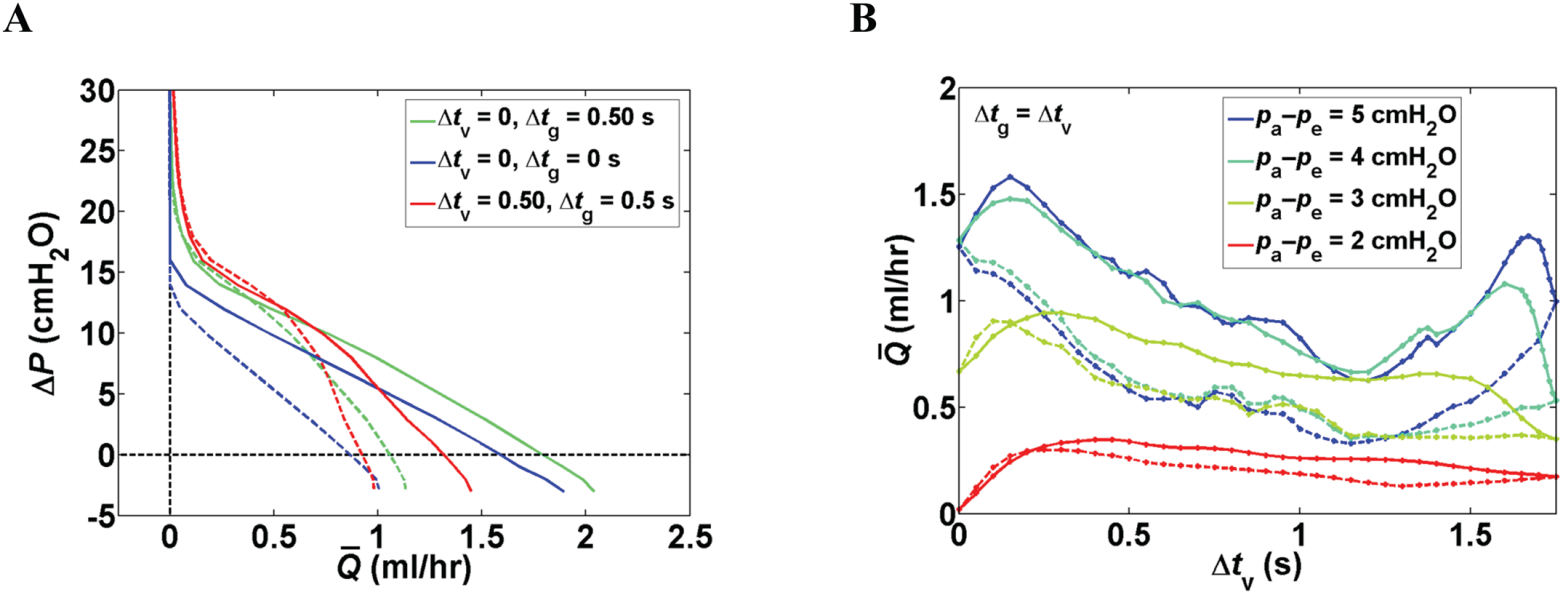
Effect of time delay between contractions of adjacent lymphangions in branching networks with *n*_v_ = 4. (A) Pump function curves for synchronized pumping (Δ*t*_g_ = Δ*t*_v_ = 0 s), time delay only at the junctions (Δ*t*_v_ = 0, Δ*t*_g_ = 0.5 s), and the baseline case (Δ*t*_g_ = Δ*t*_v_ = 0.5 s) at *p*_a_ − *p*_e_ = 4 cmH_2_O (solid lines) and *p*_a_ − *p*_e_ = 3 cmH_2_O (dashed). (B) $\bar{Q}$ vs. Δ*t*_v_ (Δ*t*_g_ = Δ*t*_v_): effect of transmural pressure via (variation of *p*_e_) at Δ*P* = 3 cmH_2_O. Solid line: forward propagating contraction wave, dashed line: reverse propagating contraction wave.

Pump function curves of branching networks with four lymphangions per vessel showed that the presence of a time delay at the junctions (Δ*t*_v_ = 0, Δ*t*_g_ = 0.5 s) is advantageous over synchronous pumping of all lymphangions (Δ*t*_v_ = 0, Δ*t*_g_ = 0). For adverse pressure differences above 10.5 cmH_2_O, additional time delay between lymphangions in one vessel (Δ*t*_v_ = 0.5, Δ*t*_g_ = 0.5 s) improved the pumping. For Δ*P* < 5.5 cmH_2_O, synchronous contractions were advantageous over the baseline case (Δ*t*_v_ = 0.5, Δ*t*_g_ = 0.5 s) (Fig 6A, solid lines). Under lower transmural pressure (achieved by elevating *p*_e_ from 2 to 3 cmH_2_O), however, synchronous contractions resulted in a less efficient pump (lower $\bar{Q}$, except for Δ*P* = –3 cmH_2_O), but addition of a time delay at the junctions (Δ*t*_v_ = 0, Δ*t*_g_ = 0.5 s) restored pumping. For Δ*P* > 6.6 cmH_2_O the highest flow rates were achieved by additional time delay between contraction of lymphangions in one vessel (Δ*t*_v_ = 0.5, Δ*t*_g_ = 0.5 s) (Fig 6A, dashed lines).

Lowering of transmural pressure (in the form of *p*_a_–*p*_e_) resulted in less pronounced peaks and valleys and a more uniform response to an incremental increase of Δ*t*_g_ = Δ*t*_v_ (Fig 6B). For a bifurcating network with *n*_v_ = 4 pumping against Δ*P* = 3 cmH_2_O, maximum $\bar{Q}$ occurred when contractions were 0.15 s apart for *p*_a_−*p*_e_ = 4 and 5 cmH_2_O (${\bar{Q}}_{\text{max}}$ = 1.47 and 1.58 ml/hr, respectively); but the flow rate response was not monotonic and $\bar{Q}$ peaked again at Δ*t*_g_ = Δ*t*_v_ ≈ 1.6 s (${\bar{Q}}_{peak}$ = 1.08 and 1.30 ml/hr). Further compressing the vessel with *p*_a_−*p*_e_ = 3 cmH_2_O produced a peak flow rate of 0.94 ml/hr at Δ*t*_g_ = Δ*t*_v_ = 0.3 s, with additional peaks at Δ*t*_g_ = Δ*t*_v_ = 1.4 and 3.4 s (= 0.1 s retrograde). Longer time delays (Δ*t*_g_ = Δ*t*_v_ = 0.45 s) were required to achieve peak $\bar{Q}$ (0.35 ml/hr) for the highest external pressure tested (*p*_a_−*p*_e_ = 2 cmH_2_O, vessels are not yet collapsed), a second lower peak was also present at Δ*t*_g_ = Δ*t*_v_ = 3.2 s (= 0.3 s retrograde) (Fig 6B). Forward propagation of contraction waves consistently produced higher flow rates. The reverse propagating wave became advantageous only in a narrow dynamic range of Δ*t*_g_ = Δ*t*_v_ < 0.16 s when *p*_a_−*p*_e_ = 3 cmH_2_O and Δ*t*_g_ = Δ*t*_v_ < 0.21 s for *p*_a_−*p*_e_ = 2 cmH_2_O. The rise in flow rate was small with a maximum increase of 0.085 ml/hr (at Δ*t*_g_ = Δ*t*_v_ = 0.05 s) and 0.04 ml/hr (at Δ*t*_g_ = Δ*t*_v_ = 0.1 s) under *p*_a_–*p*_e_ = 3 and 2 cmH_2_O, respectively.

**Fig 7:**
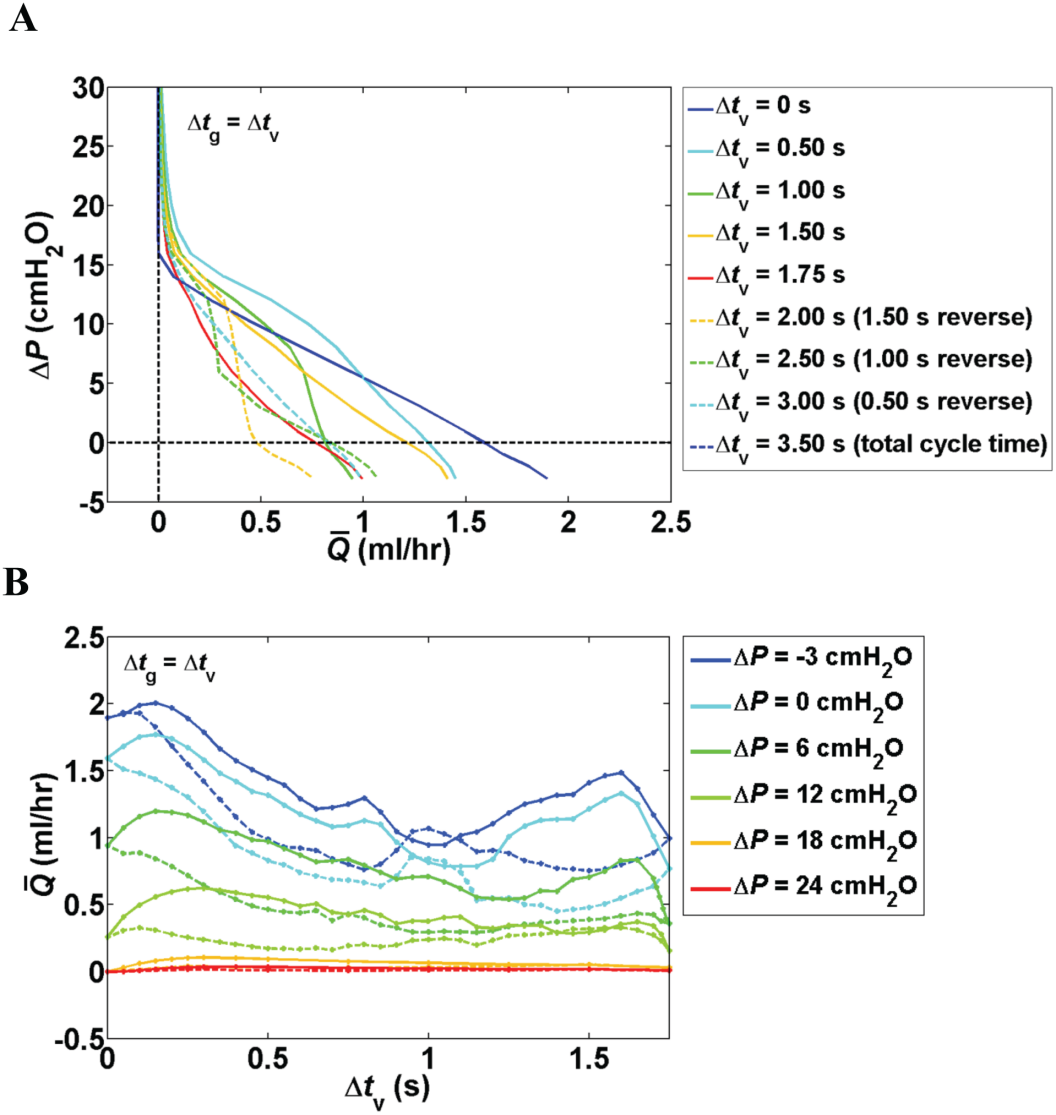
Effect of time delay between contractions of adjacent lymphangions in branching networks with *n*_v_ = 4 when Δ*t*_g_ = Δ*t*_v_. (A) Pump function curves as Δ*t*_v_ increased from 0 to 3.5 by steps of 1.0 s (Increasing *p*_b_). (B) *$\bar{Q}$*vs. Δ*t*_v_: effect of pressure difference across the network. Flow rate response as Δ*t*_v_ incrementally increased from 0 to 3.5 s. For reverse travelling waves (dashed line) equivalent time delay in the reverse direction is presented as 3.5 –(Δ*t*_g_ = Δ*t*_v_).

For branching networks with *n*_v_ = 4, pumping capability (in terms of $\bar{Q}$) increased as Δ*t*_g_ = Δ*t*_v_ increased from 0. Δ*t*_g_ = Δ*t*_v_ = 0.15 s was optimal for pumping at Δ*P* < 8 cmH_2_O (curve not shown); for higher pressure differences Δ*t*_g_ = Δ*t*_v_ = 0.25 s maximized $\bar{Q}$(curve not shown). Flow rate dropped with further time delay between contractions (larger Δ*t*_g_ = Δ*t*_v_) and the curvature of the pump function curves was altered. Δ*t*_g_ = Δ*t*_v_ = 2 s resulted in lowest flow rates under favorable pressure differences, but produced higher $\bar{Q}$ for Δ*P* > 12 cmH_2_O compared to all larger values of Δ*t*_g_ = Δ*t*_v_. Finally, at Δ*t*_g_ = Δ*t*_v_ = 3.5 s the pump function curve reverted to that with Δ*t*_g_ = Δ*t*_v_ = 0 s (total cycle time: *t*_c_ + *t*_r_ = 3.5 s) (Fig 7A).

Incremental increases of Δ*t*_g_ = Δ*t*_v_ from 0 to 3.5 s showed that under pressure differences of −3, 0, and 6 cmH_2_O, Δ*t*_g_ = Δ*t*_v_ = 0.15 s was most advantageous for pumping, resulting in peak flow rates of 2.01, 1.77, and 1.20 ml/hr, respectively. Additional peaks were also observed at Δ*t*_g_ = Δ*t*_v_ = 0.8, 1.6, 2.5 s (= 1 s retrograde) (Δ*P* = −3 and 0 cmH_2_O), and 3.4 s (= 0.1 s retrograde) (Δ*P* = −3 cmH_2_O). These regional maxima never exceeded the initial peak observed at a small non-zero Δ*t*_g_ = Δ*t*_v_. For Δ*P* = 12 and 18 cmH_2_O, Δ*t*_g_ = Δ*t*_v_ = 0.3 s resulted in peak flow rates of 0.62 and 0.11 ml/hr, respectively. The response was more uniform for larger adverse pressure differences of 18 and 24 cmH_2_O. The system was unable to pump against pressure differences of 18 and 24 cmH_2_O (Fig 7B). When the number of lymphangions *n*_v_ doubled from 4 to 8, $\bar{Q}$ still peaked for Δ*t*_g_ = Δ*t*_v_ = 0.15 s, but for *n*_v_ = 2 peak flow rate occurred with longer time delays of Δ*t*_g_ = Δ*t*_v_ = 0.25 s (figure not shown).

## Discussion

We have constructed a network scale model of contracting collecting lymphatic vessels in a symmetric branching pattern in order to understand how the characteristics of these networks determine their pumping abilities. Each vessel is itself divided into multiple lymphangions separated by lymphatic valves. This study is the first to analyze a branching network of lymphatic vessels since Reddy in the 1970s [24], and benefits from a greater understanding of lymphatic pumping, mainly from experimental work, in the intervening years. Given the highly variable patterns of coordinated lymphangion contractions in vivo, and our inability to modulate their coordination experimentally, a modeling approach is currently the best means to assess the effects of contraction coordination on pumping dynamics. Our computational approach incorporates some of the most recent measurements of parameter values and essential functional mechanisms (e.g., passive pressure diameter relationship) of these vessels, and enables us to control many of the parameters of the system that cannot be controlled experimentally. One of the main areas of focus in this study was the effect of pumping coordination in tree-like network structures on the regulation of lymph flow. This was investigated by means of a diastolic period (i.e., time delay between subsequent contractions of one lymphangion) and a time delay between contractions of adjacent lymphangions (or the conduction delay; in one vessel Δ*t*_v_ and at the junctions Δ*t*_g_). Additionally, we quantified the pumping capability of the system under increasing levels of steady transmural pressure and outflow pressure for different network sizes.

Spontaneous contractions are highly coordinated in isolated lymphatic vessels with small time delays of approximately 0–0.5 s between contractions of two adjacent lymphangions [8]. Although lymphatic vessels form highly branched networks, the complexities of *ex vivo* and *in situ* experiments pose a limit on functional studies of such structures. Our computational model of tree-like networks builds on existing data from isolated vessel experiments to explore the effects of contraction coordination on network scale behavior. We demonstrated that system output was more sensitive to the time delay at the junction Δ*t*_g_ compared to time delay between lymphangions in one vessel Δ*t*_v_. When time delay at the junction was in place (Δ*t*_g_ ≠ 0), non-zero values of Δ*t*_v_ only provided a small additional advantage under high adverse pressure differences. Thus, a small time delay only at the junctions (Δ*t*_g_ ≠ 0, Δ*t*_v_ = 0) was sufficient to maximize flow rate under a wide range of pressure differences. When transmural pressure was lowered (via higher *p*_e_) flow rate dropped but non-zero values of Δ*t*_v_ became advantageous at a lower pressure difference. At this lower transmural pressure, the presence of any form of time delay resulted in higher flow rates compared to synchronous pumping (Δ*t*_g_ = 0, Δ*t*_v_ = 0), except at the highest favorable pressure difference tested. This result could imply that under favorable pressure differences more synchrony between contractions is beneficial for lymph transport. In agreement with this finding, Davis et al. reported a 24% drop in time delay between contractions of lymphangions in rat thoracic duct when the favorable pressure difference increased from 0 to 3 cmH_2_O [9]. Thus, the effect of time delay between contractions of adjacent lymphangions on pumping efficiency is highly influenced by transmural pressure and the axial pressure difference. The advantage gained by imposing a time delay between contractions of adjacent lymphangions agrees generally with our previous studies of contracting lymphangions in series [6]. Our results further demonstrated that the change in flow rate in response to variations in outlet pressure decreased at lower transmural pressures; similar to the observations of Eisenhoffer et al. in isolated bovine mesenteric lymphatic vessels [15].

Experimental measurements of lymphangions in series *ex vivo* and *in situ* as well as branching segments (*in situ*) have confirmed the presence of both forward and reverse propagating waves of contraction from one lymphangion to another, with forward propagation appearing to be slightly more common. In the results presented here, time delays smaller than half of total cycle time (Δ*t*_g_ = Δ*t*_v_ < ½(*t*_c_ + *t*_r_)) represent forward propagating waves and larger time delays are equivalent to the reverse propagating contraction waves. For reverse travelling waves the equivalent time delay in the reverse direction can be calculated as *t*_c_ + *t*_r_–(Δ*t*_g_ = Δ*t*_v_). Overall, higher flow rates were observed with a forward propagating contraction wave relative to its equivalent reverse propagating wave. The unidirectional nature of the valves favors fluid movement in the direction of forward flow but it is not known if this directional advantage is necessarily reflected in the conduction of the electrical signal that coordinates the contractions. This is in agreement with previous modeling efforts that found forward propagating contraction waves advantageous for efficient pumping in series of lymphangions [10–12]. When all the other parameters took the baseline values, the combination of Δ*t*_g_ = Δ*t*_v_ = 0.15 s maximized pumping. Under more challenging pumping conditions (high adverse pressure difference, high external pressure) the system was less sensitive to variations in time delay. While pumping can be maximized by imposing an optimum timing between contractions of adjacent lymphangions under moderate pressure loads, these mechanisms lose their effectiveness in the presence of challenging pressure conditions.

Another determinant of pumping coordination is the diastolic period, which is the time delay between subsequent contractions of an individual lymphangion. In general, longer diastolic periods yielded higher flow rates under favorable and small adverse pressure differences. This is in agreement with recent observations in a model of lymphangions in series with transmural pressure-dependent valve behavior [12]. Additionally, we found that the lymphatic network performed less efficiently when the system was forced to pump against high adverse Δ*P*. This can be attributed to the fact that lymphatic contractions are less crucial under favorable pressure differences when the pressure head drives the fluid, and actually serve to increase impedance to incoming flow. The baseline value of *t*_r_ = 1.5 s resulted in the highest flow rate over a wide range of pressure differences. At a moderate pressure difference Δ*P* of 3 cmH_2_O, peak flow rate was achieved with longer diastolic periods as the number of lymphangions increased. Peak flow rate occurred with shorter diastolic periods as contraction frequency increased, suggesting that the ratio of diastolic period and contraction period is an influential factor. Importantly, the advantage gained (in terms of ${\bar{Q}}_{\text{max}}$) by doubling the contraction frequency was 4.7 times greater than that achieved by doubling the number of lymphangions, which shows the significance of timing of contractions for efficient pumping.

Transmural pressure is an important classical modulator of lymph transport [13, 25]. In these studies we identified a certain range of transmural pressures that maximized flow under different pressure differences and network sizes. The possibility of an optimum transmural pressure (associated with peak flow) has been suggested in previous functional studies of lymphangions in series in both experiments and modeling [14, 16, 25]. A bound on the functional range of transmural pressure is set by the fact that vessels collapse when the external pressure is higher, i.e., effective pumping requires positive transmural pressure. Then, as Δ*P* increases, flow rate decreases, but can recover to some degree with an increase in transmural pressure. In this study, flow rate maximized at lower transmural pressures when the number of lymphangions was increased. We previously showed that most effective pumping (maximum $\bar{Q}$) is achieved under transmural pressures associated with the most compliant state of the vessel on the pressure diameter curve, as it is in this range that contractions are most effective in generating stroke volume [16]. In this study, the tension generated by lymphatic muscle cells varies with diameter of the vessel and is a contributing factor as well. Thus, the most efficient pumping is achieved when there is an optimum balance of vessel compliance and contractile tension throughout the entire network. During lymphedema, accumulation of fluid in the tissue may result in higher external pressures that can collapse collecting lymphatics and inhibit effective pumping. Application of compression garments as a treatment method for this condition in some cases mitigates further limb swelling, but our results consistently indicate that application of additional external pressure actually impedes pumping in collecting vessels.

The pressure difference that the lymph pump has to overcome is another important hydrodynamic factor that regulates lymph flow. We observed that larger lymphatic networks demonstrate less variation in $\bar{Q}$ under increasing adverse pressure difference. On the other hand, larger networks perform less efficiently under favorable pressure differences. The disadvantage of larger networks stems from the additional resistances presented by longer vessels and more valves. Thus, at each level of pressure difference, it is the balance between the additional pumping power vs. increased resistance that determines whether higher flow rates can be achieved. Under adverse pressure differences, increasing the number of lymphangions generally improved pumping but a smaller advantage was gained for *n*_v_ > 4. The large number of pumping elements helped maintain the pressures that enabled the valves to remain open longer in the face of an adverse pressure difference, thus producing greater forward flow. For the pressure differences 10–15 cmH_2_O the advantage from additional pumping power is eliminated by higher resistances, which is why the curves are very close for *n*_v_ = 4–14.

There are only very few reports of experimental assessment of lymphatic pump curves, in part because the experiments are extremely difficult and subject to considerable uncertainties. Detailed comparison of modeling predications and experimental measurements is not feasible because it is impossible to measure all of the necessary pressure and flow data simultaneously to any functional degree of precision. In the few reports that do exist, similar nonlinear pump function curves have been observed experimentally [15]. The nonlinear shapes of the curves predicted by our model are the result of the complex, non-linear equations that govern the behavior of the system (e.g., pressure-diameter relationship, diameter dependent tension, valve behavior). Because many of the system parameters vary with changes in pressure and diameter, it is expected that the curves considerably change and even cross over one another.

The active and passive components of lymph transport as well as multiple regulatory mechanisms cooperate to sustain tissue fluid homeostasis. In the current computational model we described the passive behavior of the vessel using a pressure-diameter relationship obtained from isolated lymphatic vessels from rat mesentery. The tension generated during active contractions was dependent on the diameter of the vessel, to represent the physiological behavior of muscle cells. In the lymphatic system, several autoregulatory mechanisms such as stretch-dependent or shear-dependent responses modulate lymph flow by altering lymphatic muscle cell behavior (frequency and magnitude of contraction). Such regulatory mechanisms are lacking from the current model but will be included in the future refinements. In the current model we did not consider inhomogeneities in function (e.g. in contractions) or structure of the network (e.g., subsequent vessel generation via anastomoses). Experimental observations have reported regional differences in function of isolated lymphatic vessels from rat [14]. For example, mesenteric lymphatic vessels are stronger pumps whereas the thoracic duct largely relies on passive mechanisms for lymph transport [25]. Substantial differences are expected between functional aspects of lymphatic vessels in different species. Thus, obtaining more data on the biomechanical properties of human lymphatic vessels is crucial for improving the translational interpretations of this work.

In summary, we have constructed computational models of branching networks composed of contracting lymphangions. Lymph flow conditions vary in different parts of the body and under different hydrodynamic conditions. Among the many factors that influence lymph flow, we studied the effect of pumping coordination, external pressure, and axial pressure difference on lymphatic pumping. We found that the effects of these functional parameters are intertwined. For example, the dependence of the optimum timing of contractions on axial pressure difference and transmural pressure can suggest that under physiologic conditions the system uses pumping coordination as an adaptation mechanism to different levels of pressure. We confirmed that flow rate can be maximized under a certain range of transmural pressures for the network of lymphangions, in agreement with previous observations in series of lymphangions. The network scale behavior resulted in a more complex response to variations in timing of contractions between adjacent lymphangions. Inclusion of a diastolic period between the subsequent contractions of each lymphangion was advantageous for pumping, suggesting that the lymphatic system favors efficient use of metabolic energy by imposing resting periods between contractions. Additionally, the presence of this diastolic period allowed higher flow rates under a favorable pressure difference compared to the case with *t*_r_ = 0. Finally, this study provides further insight into the important factors that regulate lymph flow on a network scale. Our results demonstrate that parameters that control pumping coordination (diastolic period and time delay between contraction of adjacent lymphangions) are major determinants of effective pumping and are highly influenced by both transaxial and transmural pressure differences. Thus, efficiency of the lymphatic pump is highly dependent on the coordination of the contraction wave. Further investigation of parameters that control the direction and conduction of lymphatic pacemaking wave as well as understanding mechanisms leading to muscle contraction dysfunction are of high importance. Our results further suggest that lymphatic pumping parameters are normally interrelated and that pumping efficiency under a certain set of parameter values can vary to a large extent depending on the hydrostatic pressure conditions (transaxial and transmural pressure differences). Understanding the important mechanisms involved in muscle contraction and coordination paves the way for pharmacological treatment of underlying causes of lymphatic pumping failure in diseases like lymphedema. These findings could be potentially useful for better understanding and treatment of human lymphatic diseases but it is crucial to have better estimates on how these important pumping parameters vary in different regions of the human body.
